# Generalized Property-Based Encoders and Digital Signal Processing Facilitate Predictive Tasks in Protein Engineering

**DOI:** 10.3389/fmolb.2022.898627

**Published:** 2022-07-14

**Authors:** David Medina-Ortiz, Sebastian Contreras, Juan Amado-Hinojosa, Jorge Torres-Almonacid, Juan A. Asenjo, Marcelo Navarrete, Álvaro Olivera-Nappa

**Affiliations:** ^1^ Centre for Biotechnology and Bioengineering, Universidad de Chile, Santiago, Chile; ^2^ Departamento de Ingeniería en Computación, Universidad de Magallanes, Punta Arenas, Chile; ^3^ Max Planck Institute for Dynamics and Self-Organization, Göttingen, Germany; ^4^ Departamento de Ingeniería Química, Biotecnología y Materiales, Facultad de Ciencias Físicas y Matemáticas, Universidad de Chile, Santiago, Chile; ^5^ Escuela de Medicina, Universidad de Magallanes, Punta Arenas, Chile

**Keywords:** protein engineering, predictive models, machine learning, digital signal processing, fourier transform, numerical representation strategies

## Abstract

Computational methods in protein engineering often require encoding amino acid sequences, i.e., converting them into numeric arrays. Physicochemical properties are a typical choice to define encoders, where we replace each amino acid by its value for a given property. However, what property (or group thereof) is best for a given predictive task remains an open problem. In this work, we generalize property-based encoding strategies to maximize the performance of predictive models in protein engineering. First, combining text mining and unsupervised learning, we partitioned the AAIndex database into eight semantically-consistent groups of properties. We then applied a non-linear PCA within each group to define a single encoder to represent it. Then, in several case studies, we assess the performance of predictive models for protein and peptide function, folding, and biological activity, trained using the proposed encoders and classical methods (One Hot Encoder and TAPE embeddings). Models trained on datasets encoded with our encoders and converted to signals through the Fast Fourier Transform (FFT) increased their precision and reduced their overfitting substantially, outperforming classical approaches in most cases. Finally, we propose a preliminary methodology to create *de novo* sequences with desired properties. All these results offer simple ways to increase the performance of general and complex predictive tasks in protein engineering without increasing their complexity.

## 1 Introduction

Protein Engineering is one of the main research areas of biotechnology. It focuses on designing and implementing strategies that allow or optimize the production of proteins with desired properties. The main strategies used to achieve this objective are directed evolution and rational design. The first focuses on emulating and accelerating the evolution process, evaluating mutations and selecting those that show the desired trait, iterating the process until reaching an economically feasible optimum. The second consists of applying existing knowledge about a protein system—both empirical and theoretical—to propose mutations or variants that are likelier to exhibit the desired property.

However, predicting the outcome of replacing one (or more) amino acids in a protein sequence is a central task in Protein Engineering because it is unclear how the individual sequences relate to higher-order properties, e.g., folding Khoury et al. (2014). As protein function and properties are closely related to their constitutive amino acids (Sadowski and Jones, 2009), it is possible to design variants with enhanced functions by changing the constitutive amino acids of a sequence. However, amino acid sequences need to be *encoded* into numeric arrays in order to facilitate their computational processing. In other words, every amino acid has to be turned into a number.

Unsurprisingly, encoders play a fundamental role in the quality of the outcome of predictive models (Yang et al., 2019; Wittmann et al., 2021). However, while there is a wide variety of encoding techniques, there is no general agreement on which one to select for a specific task (Yang et al., 2018; Siedhoff et al., 2021). The first encoding approaches represented amino acid sequences in discrete manner (numeric-wise), using techniques such as One Hot or Ordinal Encoder (Winter 1998; Pavelka et al., 2009; Brownlee, 2020). However, these techniques struggle to handle high-dimensional datasets and often lack biological interpretation (Yang et al., 2018). Therefore, efficient encoding strategies allowing handling high dimensional datasets while capturing biological and physicochemical properties of the sequences are required.

Researchers have intensively used physicochemical properties of the constitutive amino acids to encode sequences (Potapov et al., 2009; Broom et al., 2017; Ancien et al., 2018). One of the open datasets summarizing these properties is the AAIndex database (Kawashima and Kanehisa, 2000), with (to date) 566 different entries for the 20 canonical amino acids. Property selection based on unsupervised machine learning (ML) algorithms (Saha et al., 2012; Forghani and Khani, 2017) often generates groups with mixed properties (Georgiev, 2009), in the sense that they are not semantically or physically coherent. Various studies have combined physicochemical properties and digital signal processing in protein engineering (Cosic and Nesic, 1987; Hejase de Trad et al., 2000). An example of such digital signal processing is the use of (Fast) Fourier Transforms (FFT) to analyze encoded sequences’ spectra. Integral transforms (as the FFT) have some interesting properties, as facilitating the convolution of signals and, eventually, capturing the interaction between amino acids. Consistently, in the context of protein engineering, transforming encoded sequences and training models in the frequency space instead allows capturing the interactions between amino acids in the whole range of the sequence (Siedhoff et al., 2020). Veljkovic et al. (1985) were pioneers in the application of discrete Fourier Transforms to analyze DNA and protein sequences. Other remarkable examples find applications in cancer studies (Cosic et al., 2016), analysis of conserved motif regions (Hejase de Trad et al., 2000), evaluation of bioactivity (Cosic, 1994), and the prediction of secondary structure and protein-protein interactions (Cosic et al., 2016). Recently, researchers have combined with great success digital signal processing with machine learning to develop predictive models to evaluate—among other variables—enantioselectivity and protein thermostability (Cadet et al., 2018a,b; Siedhoff et al., 2021).

However, the use of integral transforms (as Fourier transforms) dates further back on time (see, e.g., Eisenberg et al. (1984); Rackovsky (1998); Cosic and Nesic, 1987). Recently, Kieslich et al. aimed to generalize the property-based encoding of sequences by applying a PCA to the AAIndex. They select the 18 most explanatory principal components to define encoders and use them for training Support Vector Machine (SVM) models to predict antiviral activity on peptides, reaching outstanding performance metrics (Kieslich et al., 2021). Thereby, the authors showcase the benefits of extracting the full potential of the AAIndex dataset by proper data preprocessing. Could it then be possible to extract even more information from the AAIndex database so that we could reach higher performance metrics by employing even fewer independent encoders?

In this work, we aim to go one step beyond generalizing property-based encoding strategies and improve the numerical representation of amino acid sequences for predictive tasks in protein engineering in a way that is both explainable and consistent with previous findings. First, we applied text mining techniques to the AAIndex database to define eight semantically consistent groups of properties (i.e., groups of properties with compatible physical meaning, which naturally arise). Then, using the first component of a Kernel PCA (which is less restrictive than classic PCA), we define eight encoders that we use to represent the same protein sequence. After applying FFT, we train predictive models using the complex modulus of the Fourier spectra as input. Thereby, we facilitate the development of predictive models using ML algorithms, outperforming classical encoding strategies in the studied cases. Finally, we demonstrate the usability of the proposed approach to enhance performance in predictive tasks and to design proteins with desirable properties.

## 2 Methods

### 2.1 Semantic Clustering of Properties in the AAIndex Database

We sought to identify groups of physicochemical properties in the AAIndex database (Kawashima and Kanehisa, 2000), maximizing the separation between groups while conserving semantic consistency within them (in the sense of all properties of the same group having compatible descriptions). Our methodology combined doc2vec strategies as document representation (Kim et al., 2019) and several unsupervised learning algorithms. Below, we describe each of the four stages involved in the proposed methodology for semantic clustering and the derivation of generalized property-based encoders.

#### 2.1.1 Data Pre-Processing

We retrieved the AAIndex database records from its official site https://www.genome.jp/ftp/db/community/aaindex/aaindex1. Then, we processed the dataset generating two *.csv files to facilitate its handling, one containing numeric values for the properties and the other containing their description.

#### 2.1.2 Unsupervised Learning

As classical clustering methods based on unsupervised learning using the values of the properties cannot ensure semantic consistency within the partition generated, we designed a staged process, combining them with doc2vec techniques to generate autoencoders. We first train a doc2vec autoencoder on the descriptions of the physicochemical properties in the AAIndex database and apply it over the same dataset to obtain embedding representations. We then explore different classical unsupervised learning algorithms and combinations of their hyperparameters (as described in the exploration stage in Medina-Ortiz et al. (2020b,c)) to obtain several candidate partitions of the dataset.

#### 2.1.3 Selection of the Best Partition

We assessed the quality of each partition by obtaining their Calinsky-Harabasz indexes and selecting the one with the highest. Finally, we retrieve the original descriptions applying the inverse encoder (decoder) and review whether the condition of semantic consistency is met within the groups generated.

#### 2.1.4 Encoder Creation

Using the partition generated in the previous step, we studied how property values are distributed for the different amino acids. We created a 20 × *N*_*i*_ matrix containing the values of the *N*_*i*_ properties contained in the *i* − th group for each amino acid. We then applied a kernel principal component analysis (kernel-PCA, radial basis function RBF-kernel with default settings) to the matrix representing each group. Noteworthy, a kernel-PCA expands the traditional PCA’s limitations, such as requiring the components (namely, columns of the matrix) to distribute normally, and prevents the information loss that would cause removing those properties that do not meet this condition. Finally, we define encoders as the first component of each intra-group kernel-PCA.

### 2.2 Numerical Representation of Protein Sequences and Fast Fourier Applications

The general principle behind encodings is to map a categorical variable into a numeric value. In the context of protein engineering, encoding sequences of amino acids translates them into vectors. However, distance-based algorithms cannot capture the interactions between residues when comparing different sequences (or variants of the same sequence when replacing one or more of the constitutive amino acids). As we expect changes in one residue to impact the protein’s function depending on who the neighbor residues were, we need a method to account for the impact that each amino acid has on the whole sequence. One way to capture this broad range of interactions is to use Fourier transforms (Sneddon, 1995).

Alongside other integral transforms, Fourier transforms search to represent functions (or vectors) as a superposition of other functions or vectors that form a basis of the correspondent space. For Fourier transforms, such a basis is all possible sinusoidal functions. Although it was originally thought for a continuously valued function, it is possible to define the Fourier transform and its inverse for discrete distributions. In this case, only a finite sample segment of the continuous data set is required to reconstruct the frequency spectrum (Rao and Yip, 2014).

The Fast Fourier transform (FFT) algorithm enables the efficient computation of the Fourier transform; Solving the problem directly from the discrete Fourier Transform (DFT) yields a complexity of *O*(*N* ^2^), while using the FFT generates a complexity of *O*(*N* log  *N*) (Welch, 1967). In the context of the present work, we apply FFT to each encoded sequence according to the following steps: 1) As required to apply FFT, we complete every vector with zeros (zero padding) such that the resulting dimension is (2^*n*^) − 1. 2) We apply FFT to each resized vector independently, obtaining a $n\times \frac{m}{2}$ matrix of frequencies, where *n* is the number of sequences and *m* is the number of points in the vector. We then use the obtained frequencies as input to train predictive models.

### 2.3 Predictive Models Training

Throughout the different case studies presented in this work, we use Random Forest predictive models due to their easy implementation and interpretation. Hyperparameters are those of the default configuration of DMAKit (Medina-Ortiz et al., 2020b): n_estimatorsint = 100, criterion = gini, min_samples_split = 2, min_samples_leaf = 1, and n_jobs = −1 so that all available cores are used. After preprocessing, each input dataset was divided into training and testing datasets in an 80:20 ratio. For the performance assessment experiment, we repeated the 80:20 split of the dataset 1.000 times using different random seeds, aiming to compensate for any potential selection bias. Thus, instead of reporting a single value for model precision, we report, in this case, a distribution. Model training involves a k cross-validation stage, with k = 10. We also put forward a metric to assess overfitting, the overfitting ratio, defined as the ratio between model precision in the training and validation stages.

### 2.4 Testing Datasets and Case Studies

Here we describe the different datasets we evaluated in the case studies to assess the proposed encoders and methodology.

#### 2.4.1 DNA-Binding Protein

DNA-binding protein (DBP) classification is one of the most exciting problems in biotechnology, mainly because of its implications in protein engineering, synthetic biology, molecular biology, and genetic engineering (Rahman et al., 2018). Furthermore, it finds direct application in the improvement of commercial DNA polymerases and restriction enzymes (Wei et al., 2017). Different computational methods to develop classification models for DNA-Binding protein have been proposed, involving various sequence coding and characterization strategies. Despite the enormous efforts aiming to solve this problem, it remains open. The dataset for this task was built using different previously reported datasets (Wei et al., 2017; Rahman et al., 2018; Adilina et al., 2019). We also removed all sequences without classification, generating a balanced dataset with 504 examples of DNA binding protein and 523 non-DNA binding protein.

#### 2.4.2 Folding and Function Recognition

Two of the most common tasks in protein engineering are the prediction of the folding of secondary structures and the classification of protein function (Marchler-Bauer et al., 2017). Based on this, our approach was based on solving two questions of interest. 1) Given a set of proteins with the same folding, is it possible to recognize or predict the functions of these proteins? 2) Given a set of proteins with the same function, is it feasible to classify protein folding? First, we used the Protein Data Base (PDB) to build these data sets. Then, for each search, we applied the following filters: 1) *Homo sapiens* organism, 2) X-Ray diffraction as the experimental method, 3) Protein type as Polymer entity type, and 4) a resolution lower than 3 Å. Next, we implemented a bash script to download the protein sequences and save them in csv files for the different applications. Remarkably, we developed balanced datasets to reduce the possible problems in the training process in all cases.

#### 2.4.3 Biological Activity Prediction for Peptide Sequences

Antimicrobial peptides (AMPs) are known as host-defense peptides (Sitaram and Nagaraj, 2002). These molecules play an essential role in the innate immune response, thus having direct application in the pharmaceutical, biotechnological, and industrial areas (Papagianni, 2003; Ma et al., 2018). Different computational methods based on ML have been developed to classify antimicrobial peptides (Xiao et al., 2013; Chen et al., 2016; Zimmer et al., 2018; Yi et al., 2019; Yi et al., 2019). In this case study, we used the peptide sequences reported in PeptipediaDB (Quiroz et al., 2021) to develop classification models of AMPs peptides, generating a dataset with six types of biological activities.

### 2.5 Implementation Strategies and Library Developing

Scripts to develop, assess, and exemplify the usage of the proposed encoders are written in *Python* v3.9, powered by libraries as Pandas (McKinney, 2010), Numpy, Gensim (Řehřek and Sojka, 2011), and DMAKit (Medina-Ortiz et al., 2020b), among others. The encoding library proposed in this work was designed under the Object-Oriented Programming paradigm (Wegner, 1990), which is advantageous for its modularity.

## 3 Results and Discussion

### 3.1 Combining Text Mining and Unsupervised Learning Reveal Semantic Groups of Physicochemical Properties in the AAIndex Database

Using a combination of doc2vec strategies and unsupervised learning algorithms, we identified eight groups semantically-consistent groups of physicochemical properties within the AAIndex database (Kawashima and Kanehisa, 2000). By semantic consistency, we refer to these groups representing the same physical aspect of amino acids, such as general structural and thermodynamic properties and indices. To determine them, we explored about one million possible partitions of the dataset, changing the way of generating embeddings of property descriptions, the clustering algorithms, and their hyperparameters. We performed this using the model exploration tools presented in Medina-Ortiz et al. (2020b).

The resulting eight groups of properties were obtained by training autoencoders with hyperparameters of 500 epochs, a value of *α* = 0.025, and an embedding size of 2, and partitioning the dataset by applying the *k* − means algorithm with *k* = 8. This was the best performing algorithm found in the exploration stage, reaching a Calinski-Harabasz index of **1,532.36** and a silhouette coefficient of **0.43**. Finally, we assessed the semantic consistency of each group, evaluating whether the properties within the group presented the same contexts or specific words. As a result, only 17 descriptions were reclassified from the group of *Other indexes* to the groups of *α* *structure* and *β* *structure*.

One of the advantages of implementing a strategy based on doc2vec is the semanticity generated by separating the properties by their descriptions, which facilitates a simple visualization of the existing contexts or topics in each group. On the other hand, applying unsupervised learning algorithms to property values will generate partitions that do not ensure semantic consistency within groups, as clustering criteria will be numeric. In this way, the semantic clustering methodology proposed in this work ensures that the random selection of any member of a particular group will have the same physical meaning, otherwise not possible.

We analyzed the AAIndex database from a numeric perspective to test the statement above. We explored different combinations of unsupervised learning algorithms and hyperparameters to partition the dataset. The best performing algorithm was *k* − means (*k* = 2), yielding a Calinski-Harabasz index of **1,527.81** and a silhouette coefficient of **0.87**. Although these results hint at an excellent separation between the groups in the partition, not only is there no relationship between the descriptions within the groups, but also unbalanced divisions of properties between groups. Forcing the *k* − means algorithm to produce **eight** groups generates a partition with a Calinski-Harabasz index of **614.25** and a silhouette coefficient of **0.50**. However, and as expected, no semantic consistency within the groups was achieved.

**FIGURE 1:**
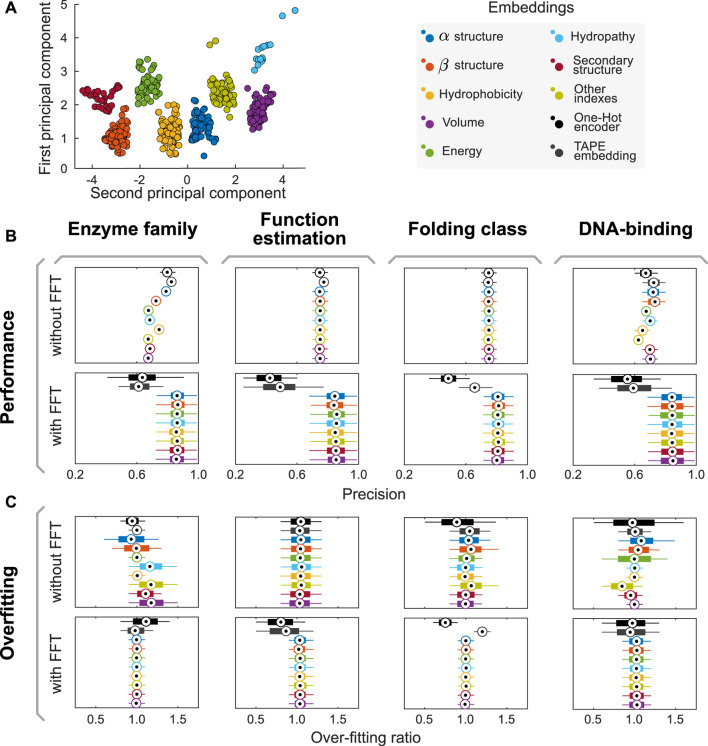
The AAIndex database of amino acid physicochemical properties can be split into eight semantically-consistent groups. **(A)** Combining doc2vec strategies with unsupervised learning algorithms, we proposed a methodology to generate groups that preserve semantic consistency within the partition. Applying an RBF kernel PCA on the whole dataset, we observe that the groups are linearly separable in the PCA1/PCA2 space, as their convex hulls are disjoint. **(B,C)** Combining our encoders with FFT improves model performance and helps reducing overfitting in several predictive tasks. Here, boxplots summarize the distribution of performances reached in each experiment across the 1,000 independent realizations of the 80/20 split of the input dataset for the task. Central circles represent medians, bars the interquartile range, and whiskers the 95% CI. Complementary analyses of model performance, including other metrics (such as recall, F-Score, and area under the receiver operating curves AUC), are presented in Supplementary Section S3 and summarized in Supplementary Tables S3–S6.

Once the groups of descriptions were generated and corrected, these were used to generate eight data sets with the property values for each amino acid. We applied a kernel-PCA (Radial Basis Function–RBF–kernel) to the numeric values of each group and assessed how much of the variance was explained by the first component. In all groups, the variance explained by the first component of the kernel-PCA was higher than **85%**. Furthermore, the different groups resulted in being linearly separable in the PCA1/PCA2 space, as their convex hulls are disjoint (cf. Figure 1A). Therefore, we proposed to use the first component of each semantic group of properties generated as an encoder. These encoders are listed in Table 1.

**TABLE 1:** Generalized property-based encoders for amino acids.

Amino acid	*α* structure	*β* structure	Hydrophobicity	Volume	Energy	Hydropathy	Secondary structure	Other indexes
A	290.41	71.85	6.25	44.65	−107.79	15.33	56.16	92.92
R	172.57	−6.96	84.09	200.15	51.15	172.36	1.44	−37.39
N	−38.37	−90.14	−21.73	−191.18	73.94	−259.13	−54.69	−77.74
D	159.43	−56.58	−28.96	−232.26	55.36	−216.01	−29.38	−7.42
C	−4.24	15.67	−34.88	−156.21	−54.19	−242.01	10.07	40.04
Q	−268.55	−32.61	38.46	179.88	31.44	145.73	-15.43	−45.52
E	−0.02	21.03	−21.48	−170.44	−49.97	8.11	20.20	50.74
G	−104.49	−62.33	53.16	250.66	92.25	256.52	-39.89	−95.41
H	−159.87	31.27	−69.67	194.47	−39.54	455.61	34.12	43.37
I	−34.08	164.64	−54.85	−88.56	−48.44	−274.76	25.05	52.40
L	−91.11	−16.38	−64.98	−201.08	7.56	−257.27	−10.20	4.27
K	195.59	54.45	−52.92	−118.84	−109.99	−136.28	55.31	85.66
M	21.94	−18.77	−26.70	−227.61	−7.39	−139.71	−19.45	16.04
F	88.02	21.61	−21.46	−78.96	−56.97	80.68	30.31	46.42
P	317.10	115.37	−22.23	−44.80	−157.63	−126.45	95.69	136.09
S	−314.20	−106.56	61.31	221.12	174.08	248.05	−85.57	−122.66
T	−252.51	−23.99	13.72	−3.30	17.50	−153.13	−25.56	−31.46
W	−118.15	−76.02	88.28	34.80	105.47	19.24	−59.91	−124.49
Y	−10.20	−15.49	40.85	203.07	36.61	171.61	−4.25	−33.07
V	150.75	9.929	33.77	184.45	−13.45	231.50	15.99	7.21

### 3.2 Semantically-Consistent Encoders and Fourier Transform Facilitate Predictive Tasks in Protein Engineering

We used the proposed semantic encoders to tackle four different predictive tasks in protein engineering (DNA-binding protein classification, protein folding, protein function, and enzyme family determination) using Random Forest algorithms. The datasets and hyperparameters are described in the Methods section. First, input datasets were split into training and validation datasets in an 80:20 proportion. Then, aiming to prevent any stochastic artifact induced by a favorable/unfavorable partition of the dataset, we repeated this stage 1,000 times using different random seeds. Thus, instead of obtaining a single value for the performance of a model, we obtained a distribution of performances (cf. Figures 1B,C). When comparing model performance achieved using our encoders with that of models trained with classical methods (e.g., One Hot Encoder (Broom et al., 2017) and TAPE embeddings (Rao et al., 2019)), there is no major difference (cf. Figure 1B). However, our models reached over-fitting ratios (defined as the performance of training divided by the performance in validation) closer to one than classical approaches, suggesting that our encoders are better suited for these predictive tasks (cf. Figure 1C).

We repeated the experiments above but applied the Fast Fourier Transform (FFT) to the encoded sequences before training predictive models. We then use the complex modulus of the discrete Fourier transform as a feature to train our models. By doing so, we aim to capture the influence of the position of each amino acid within the sequence, which affects other amino acids in different ranges of influence. We will provide further details on the interpretation of the FFT-related variables in the next section. While there is a drop in performance when combining One Hot and embedding-based encoders and FFT, the use of FFT increased the precision of predictive models trained using the semantic encoders herein proposed. Moreover, the over-fitting ratio decreases even further in this case, suggesting a synergistic effect on the predictive performance of trained models.

A possible interpretation of this effect relates to the Fourier transform’s properties, which capture the influence of each component of the input on the others, thereby incorporating more information into the predictive systems. Amino acids within a protein sequence influence each other. Thus, by applying Fourier Transforms, we can capture, to some extent, this spatial dependency. Furthermore, this property results beneficial for any property-based encoding strategy, as previously reported in Siedhoff et al. (2021), Cadet et al. (2018b), and Cosic (1994). Based on the above, we propose the combination of our encoders together with the application of Fourier transforms in order to improve the performance of predictive models.

We performed a complementary model evaluation analyzing the whole spectra of performance metrics, including recall, F-Score, and area under the receiver operating curves AUC. We found a marked consistency between the precision and recall obtained by trained models, and these metrics were further increased when training models in the frequency space. Furthermore, the high values reached for the AUC across predictive tasks highlight the predictive power of our approach. The reader is referred to Supplementary Section S3, and Supplementary Tables S3–S6.

We compared the performance of our encoders and similar approaches to assess whether we reached a sweet spot regarding the number of proposed encoders and information contained therein. In particular, we compare our results against 1) using all properties in the AAIndex as independent encoders and 2) applying a linear PCA directly on the AAIndex database and using the most informative components as independent encoders. The reader is referred to Supplementary Section S4.

### 3.3 The Combination of Our Encoders With FFT Allow Detecting Profiles Related to Folding and Protein Functions

**FIGURE 2:**
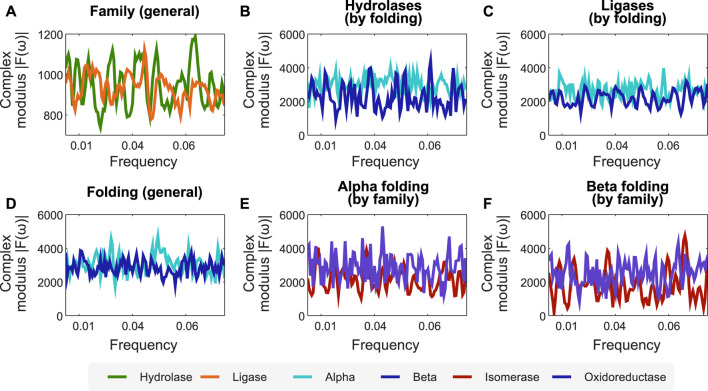
The combination of our encoders with FFT unveils frequency profiles associated to specific protein folding and functions. We used the encoder of secondary structure combined with FFT to create profiles related to folding and protein functions. **(A)** Fourier spectra for two family enzymes (hydrolases and ligases) in a dataset of enzyme families. **(B,C)** Fourier spectra of the same family separated by folding, showing that our methodology is sensitive to apparent differences between alpha and beta folding types. **(D)** Fourier spectra for alpha and beta folding in a dataset of different protein families. **(E,F)** Fourier spectra of the same folding separated by protein family, showing that our methodology is sensitive to proteins with the same folding but belonging to different families. *N* for frequency normalization = 1,024.

Combining the encoders proposed in this work, the interpretation of protein sequences as signals, and processing them after applying FFT, facilitates the identification of profiles at the folding and functional levels. To demonstrate this, we propose the following case study. We encode the *protein function* dataset employing the *secondary structure*-related encoder and apply FFT on it to analyse its spectra. In particular, we sought to find relations for the mean complex modulus of the transformed signals of different families of enzymes (in this case, hydrolase and ligase), and used their length to x-scale the frequency, zoomed to the active site, and excluded extremes that could be affected by either zero-padding or border conditions. We found a clear difference between the mean complex modulus of the Fourier spectra of ligases and hydrolases (cf. Figure 2A). Furthermore, we also found differences in the mean complex modulus of the Fourier spectra when analyzing two folding classes within the same family (cf. Figures 2B,C).

We employ the same approach (*secondary structure*-related encoder combined with FFT) to identify protein folding profiles and sub-profiles related to protein function. Figure 2D shows the average spectra for the *α* and *β* folds of enzymes with different functions. Similarly, we found that isomerases and oxidoreductases have slightly different mean Fourier spectra, although sharing the *α* and *β* folding properties (cf. Figures 2E,F).

### 3.4 Towards a New Design Strategy for Protein Sequences With Desirable Properties

**FIGURE 3:**
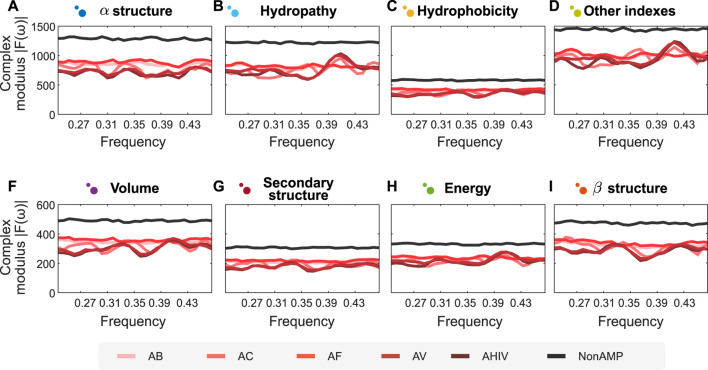
Fourier spectra of encoded amino acid sequences with different activities are visually separated. Sub figures show the Fourier spectrum of different sequences of peptides, encoded according to the groups of properties proposed in this article, represented in panels **(A–I)**. We analyse two types of peptides: Antimicrobial (AMPs) and non-Antimicrobial (nonAMP). AMPs are subsequently divided into five categories: Antibacterial Peptides (AB), Anticancer Peptides (AC), Antifungal Peptides (AF), Anti-HIV Peptides (AHIV), and Antiviral Peptides (AV). The signals analyzed show a clear differentiation for AMPs concerning nonAMPs. *N* for frequency normalization = 128.

One of the most challenging problems in protein engineering is protein design (Yang et al., 2019). Considering the advantages of combining our semantic encoders and FFT, we put forward a prospective methodology to design peptide sequences with desired properties. In this case study, we illustrate the use of this methodology to design peptides with antimicrobial activity. Using the antimicrobial peptide dataset described in Methods, we apply our encoders and FFT to the dataset and trained two random forest predictive models. The first model is a binary classification model for antimicrobial activity, while the second corresponds to a multi-class model of various biological activities for antimicrobial peptides. The latter include peptide classes such as antibacterial, anti-viral, anti-cancer, anti-HIV, and anti-fungal. The models had an accuracy of respectively **95.3%** and **89.41%**. On the one hand, the clear separation between the spectra of antimicrobial and non-antimicrobial peptides explains the high performance reached by the binary classifier. On the other hand, marked patterns for each biological activity facilitate the generalization in the multi-class model (cf. Figure 3, where panels A–I represent the different encoders proposed herein). Altogether, when analyzing the distribution of values for each position, we can define a *latent space* where, theoretically, encoded signals with the same complex modulus would have the same activity.

After characterizing the classification mechanisms of the models described above, we put forward the following methodology to generate new sequences that would be classified as “having an activity” by them. First, we collect different peptide sequences with antimicrobial activity from the Peptipedia database (Quiroz et al., 2021). These sequences are new examples for the classifiers, as they were not used during the model training step. Alternatively, another way to generate new sequences for this stage is through deep generative models (Wu et al., 2021). Note that we already know that these sequences do have antimicrobial activity. These build up a *m* × *n* matrix, where *m* are the number of sequences and *n* the length of the longest of those (all others are completed with zeros). Second, we encode and transform the sequences using all the proposed encoders in this work separately to obtain 8 *m* × *n* matrices. Third, we characterize the distribution of values column-wise for each matrix, so we obtain confidence intervals for the encoded values of each position. Fourth, we calculate the likelihood of new sequences belonging to each category’s latent space for each encoder. Precisely, for each residue in the sequence, we calculate a probability. Assuming that all these are independent, the t probability of belonging to the latent space is the multiplication of the individual probabilities obtained for each position. In this way, we have eight statistical tests where belonging to a latent space could predict a unique biological activity. Fifth, we used the trained model to predict the category of the proposal sequence. Finally, we evaluate the predictions and check if the proposed sequences are classified in the class of interest. A step-by-step, in-depth explanation of the proposed methodology and a summary flowchart can be found in Supplementary Section S4.

Using the proposed strategy, we randomly explored 10,000 sequences. We defined a selection criterion of 90% probability of existing within the latent space of desirable biological activity, in this case, antimicrobial peptides and their different subcategories. Of the 10,000 sequences explored, only **3,513** met the established probability criteria, and their activity was predicted using the previously trained models. Remarkably, because the biological activities of the sequences were known in advance, the performance of the screening methodology could be evaluated by comparing the predicted rankings with the biological activities reported by each sequence. Performance metrics are reported in Supplementary Table S3. Notably, the sequences recognized as antimicrobial peptides showed performance similar to the training result. However, the rest of the biological activities evaluated showed a decrease concerning the predictive model. This is not surprising since the models were trained using sequences that only presented a specific activity, while the evaluated sequences showed primarily moonlight activity (which is why the sub-activities of antimicrobial peptides do not add up to the total number of antimicrobial peptides evaluated sequences). Despite these results, the proposed methodology facilitates the exploration of new sequences from a probabilistic point of view, being enormously efficient for antimicrobial peptides and promising for future applications.

## Conclusion

The results presented in this work can be summarized as three main contributions. First, we extend the traditional property-based encoding strategy and propose eight new encoders that represent semantically-consistent groups of physicochemical properties of the AAIndex database. Second, we illustrate how using these encoders together with Fourier transforms can substantially improve the performance of machine learning models in general predictive tasks in protein engineering. Furthermore, we found a synergistic interaction between the proposed encoders and the FFT that simultaneously increases the precision of the trained models while reducing their overfitting to the data. Finally, we put forward a simple and preliminary statistically-based methodology to create *de novo* peptide and protein sequences with desirable properties. We will extend the modeling framework to simultaneously use the eight encoders to tackle more complex predictive tasks in protein engineering in future work. We expect these independent descriptions of a sequence to interact synergistically and increase model performance.

## Data Availability

The original contributions presented in the study are included in the article/Supplementary Material, further inquiries can be directed to the corresponding authors.
