# Supplementary material for: Generalized Property-Based Encoders and Digital Signal Processing Facilitate Predictive Tasks in Protein Engineering
Source: Front Mol Biosci. 2022 Jul 14;9:898627. doi: 10.3389/fmolb.2022.898627 (PMC9329607; doi:10.3389/fmolb.2022.898627)
Supplement: Supplementary file 1 [file DataSheet1.PDF]

# Generalized property-based encoders and digital signal processing facilitate predictive tasks in protein engineering

Supplementary Information

## S1 Summary of explained variance for each Kernel PCA applied

| Cluster-ID | **Property Keyword** | **# elements** | **Variance** ($1^{st}$ Component) |
|---|---|---|---|
| ID-01 | $\alpha$ structure | 97 | 91.13% |
| ID-02 | $\beta$ structure | 58 | 86.57% |
| ID-03 | Energy | 82 | 87.36% |
| ID-04 | Hydropathy | 61 | 96.03% |
| ID-05 | Hydrophobicity | 27 | 86.16% |
| ID-06 | Other indexes | 69 | 85.13% |
| ID-07 | Secondary structure | 237 | 87.35% |
| ID-08 | Volume | 61 | 87.71% |

Table S1: Description of the groups of properties obtained applying the methodology proposed in this work to the AAIndex database.

## S2 Dataset descriptions

| **Dataset ID** | **Description** | **Type file** | **# Examples** |
|---|---|---|---|
| DS-01 | Anti microbial peptide classification | Fasta | 1832 |
| DS-02 | Biological activity for peptide sequences multi task | Fasta | 3796 |
| DS-03 | DNA Binding protein classification | CSV | 1027 |
| DS-04 | Enzyme Family classification | CSV | 1827 |
| DS-05 | Folding classification | CSV | 3132 |
| DS-06 | Function Classification | CSV | 3132 |

Table S2: Summary generated datasets to test the proposed methodology

## S3 Summary performance for protein engineering encoding tasks

In this section, we complement the analysis presented in Figure 1 of the main text. In particular, we analyze the whole spectra of performance metrics (i.e., precision, recall, F-score, and area under the receiver operating curves AUC) of the predictive models in the different tasks analyzed. In the case of multi-class models, we estimate the AUC as the weighted average for each category. In most cases, there is a marked consistency between the values obtained for precision and recall and high AUCs across tasks, verifying the robustness and predictive power of the approach. Furthermore, the increase in performance when applying FFT as

preprocessing is consistent for all metrics, displaying the synergy between the proposed encoders and this kind of integral transformation. Tables S3, S4, S5, and S6 summarize the estimated performances.

| Applying FFT? | Encoder | Precision | Recall | F-score | AUC |
|---|---|---|---|---|---|
| No | Alpha structure | 0.749 | 0.787 | 0.725 | 0.719 |
| | Beta structure | 0.763 | 0.761 | 0.721 | 0.719 |
| | Hydrophobicity | 0.667 | 0.761 | 0.723 | 0.718 |
| | Volume | 0.776 | 0.706 | 0.750 | 0.744 |
| | Energy | 0.657 | 0.674 | 0.724 | 0.707 |
| | Hydropathy | 0.777 | 0.767 | 0.733 | 0.726 |
| | Secondary Structure | 0.728 | 0.664 | 0.707 | 0.746 |
| | Other Indexes | 0.767 | 0.761 | 0.723 | 0.721 |
| Yes | Alpha structure | 0.871 | 0.819 | 0.853 | 0.808 |
| | Beta structure | 0.847 | 0.874 | 0.818 | 0.805 |
| | Hydrophobicity | 0.846 | 0.880 | 0.820 | 0.897 |
| | Volume | 0.874 | 0.893 | 0.843 | 0.823 |
| | Energy | 0.846 | 0.887 | 0.823 | 0.895 |
| | Hydropathy | 0.873 | 0.867 | 0.830 | 0.829 |
| | Secondary Structure | 0.862 | 0.806 | 0.841 | 0.823 |
| | Other Indexes | 0.875 | 0.819 | 0.855 | 0.899 |

Table S3: Summary performance for validation step in DNA-Binding classification task

| Applying FFT? | Encoder | Precision | Recall | F-score | AUC |
|---|---|---|---|---|---|
| NO | Alpha structure | 0.780 | 0.775 | 0.772 | 0.962 |
| | Beta structure | 0.790 | 0.786 | 0.782 | 0.964 |
| | Hydrophobicity | 0.784 | 0.784 | 0.781 | 0.962 |
| | Volume | 0.774 | 0.773 | 0.770 | 0.967 |
| | Energy | 0.798 | 0.796 | 0.792 | 0.96 |
| | Hydropathy | 0.774 | 0.775 | 0.771 | 0.963 |
| | Secondary structure | 0.779 | 0.779 | 0.775 | 0.961 |
| | Other indexes | 0.781 | 0.777 | 0.773 | 0.962 |
| YES | Alpha structure | 0.889 | 0.890 | 0.886 | 0.970 |
| | Beta structure | 0.794 | 0.793 | 0.789 | 0.971 |
| | Hydrophobicity | 0.895 | 0.892 | 0.889 | 0.971 |
| | Volume | 0.898 | 0.894 | 0.891 | 0.972 |
| | Energy | 0.893 | 0.892 | 0.888 | 0.870 |
| | Hydropathy | 0.898 | 0.895 | 0.890 | 0.968 |
| | Secondary structure | 0.893 | 0.893 | 0.889 | 0.970 |
| | Other indexes | 0.892 | 0.894 | 0.890 | 0.970 |

Table S4: Summary performance for validation step in enzyme family classification task

# S4 Comparison of predictive models using properties of AAIndex database

This section presents a complimentary benchmark analysis where we compare our results with similar approaches. Inspired by the works of [Cadet et al.(2018a)Cadet, Fontaine, Li, Sanchis, Chong, Pandjaitan et al., Cadet et al.(2018b)Cadet, Fontaine, Vetrivel, Chong, Savriama, Cadet et al., Siedhoff et al.(2021)Siedhoff, Illig, Schwaneberg, Kieslich et al.(2021)Kieslich, Alimirzaei, Song, Do, and Hall], we propose the following experiment. First, we used all properties in the AAIndex database (n=553 after filtering empty entries) to train independent models,

| Applying FFT? | Encoder | Precision | Recall | F-score | AUC |
|---|---|---|---|---|---|
| NO | Alpha structure | 0.772 | 0.757 | 0.722 | 0.973 |
| | Beta structure | 0.786 | 0.774 | 0.739 | 0.977 |
| | Hydrophobicity | 0.769 | 0.764 | 0.737 | 0.978 |
| | Volume | 0.801 | 0.796 | 0.766 | 0.976 |
| | Energy | 0.777 | 0.781 | 0.749 | 0.968 |
| | Hydropathy | 0.797 | 0.7964 | 0.767 | 0.979 |
| | Secondary structure | 0.786 | 0.787 | 0.760 | 0.976 |
| | Other indexes | 0.777 | 0.773 | 0.7407 | 0.977 |
| YES | Alpha structure | 0.863 | 0.855 | 0.840 | 0.975 |
| | Beta structure | 0.865 | 0.857 | 0.841 | 0.971 |
| | Hydrophobicity | 0.806 | 0.806 | 0.784 | 0.975 |
| | Volume | 0.862 | 0.857 | 0.842 | 0.972 |
| | Energy | 0.871 | 0.861 | 0.848 | 0.975 |
| | Hydropathy | 0.878 | 0.863 | 0.848 | 0.974 |
| | Secondary structure | 0.871 | 0.861 | 0.848 | 0.978 |
| | Other Indexes | 0.879 | 0.870 | 0.859 | 0.976 |

Table S5: Summary performance for validation step in folding classification task

| Applying FFT? | Encoder | Precision | Recall | F-score | AUC |
|---|---|---|---|---|---|
| NO | Alpha structure | 0.874 | 0.870 | 0.871 | 0.973 |
| | Beta structure | 0.877 | 0.876 | 0.876 | 0.973 |
| | Hydrophobicity | 0.879 | 0.876 | 0.876 | 0.975 |
| | Volume | 0.883 | 0.880 | 0.881 | 0.974 |
| | Energy | 0.875 | 0.872 | 0.873 | 0.976 |
| | Hydropathy | 0.877 | 0.873 | 0.874 | 0.973 |
| | Secondary structure | 0.871 | 0.869 | 0.870 | 0.973 |
| | Other indexes | 0.881 | 0.878 | 0.879 | 0.974 |
| YES | Alpha structure | 0.888 | 0.885 | 0.886 | 0.976 |
| | Beta structure | 0.890 | 0.886 | 0.887 | 0.977 |
| | Hydrophobicity | 0.889 | 0.886 | 0.887 | 0.976 |
| | Volume | 0.889 | 0.887 | 0.888 | 0.975 |
| | Energy | 0.891 | 0.887 | 0.888 | 0.975 |
| | Hydropathy | 0.887 | 0.883 | 0.884 | 0.975 |
| | Secondary structure | 0.888 | 0.883 | 0.884 | 0.975 |
| | Other indexes | 0.887 | 0.884 | 0.885 | 0.976 |

Table S6: Summary performance for validation step in function classification task

with and without applying FFT in a preprocessing stage. Second, we applied a linear PCA directly to the AAIndex database, selected the most informative components (n=20), and used them to train independent models. Again, the above with and without FFT. In summary, we have the following combinations:

- Using each property in the AAIndex as independent encoders.
- Using each property in the AAIndex as independent encoders, and apply FFT to encoded sequences.
- Using the 20 most informative components of a linear PCA applied to the AAIndex as independent encoders.
- Using the 20 most informative components of a linear PCA applied to the AAIndex as independent encoders, and apply FFT to encoded sequences.

We applied a Random Forest algorithm to train predictive models employing a division of 80:20 to create training and validation datasets. Also, we apply a $k-$fold cross-validation with $k = 10$ over the training data

to prevent overfitting. We use the precision as standard performance metric. The results are summarized in the Tables S7, S8, S9, and S10.

| Task | Strategy | Performance | Max value | Min value | Average | Standard deviation |
|---|---|---|---|---|---|---|
| DNA-Binding protein classification | Only properties | Training | 0.671 | 0.604 | 0.635 | 0.012 |
| | Only properties | Validation | 0.711 | 0.605 | 0.665 | 0.016 |
| | Only properties | Over-fitting rate | 1.062 | 0.869 | 0.955 | 0.027 |
| | Only properties (+FFT) | Training | 0.675 | 0.588 | 0.636 | 0.014 |
| | Only properties (+FFT) | Validation | 0.713 | 0.611 | 0.666 | 0.016 |
| | Only properties (+FFT) | Over-fitting rate | 1.031 | 0.884 | 0.955 | 0.027 |
| | Linear PCA encoders (20) | Training | 0.664 | 0.511 | 0.6348 | 0.031 |
| | Linear PCA encoders (20) | Validation | 0.709 | 0.252 | 0.650 | 0.092 |
| | Linear PCA encoders (20) | Over-fitting rate | 2.028 | 0.890 | 1.009 | 0.236 |
| | Linear PCA encoders (20) (+FFT) | Training | 0.654 | 0.511 | 0.628 | 0.029 |
| | Linear PCA encoders (20) (+FFT) | Validation | 0.688 | 0.252 | 0.643 | 0.090 |
| | Linear PCA encoders (20) (+FFT) | Over-fitting rate | 2.028 | 0.919 | 1.009 | 0.234 |

Table S7: Summary of statistical values for exploration properties with different strategies on dna-binding protein classification

| Task | Strategy | Performance | Max value | Min value | Average | Standard deviation |
|---|---|---|---|---|---|---|
| Enzyme classification | Only properties | Training | 0.801 | 0.780 | 0.791 | 0.003 |
| | Only properties | Validation | 0.797 | 0.760 | 0.776 | 0.005 |
| | Only properties | Over-fitting rate | 1.040 | 0.991 | 1.018 | 0.008 |
| | Only properties (+FFT) | Training | 0.816 | 0.775 | 0.801 | 0.005 |
| | Only properties (+FFT) | Validation | 0.809 | 0.753 | 0.791 | 0.007 |
| | Only properties (+FFT) | Over-fitting rate | 1.045 | 0.983 | 1.012 | 0.009 |
| | Linear PCA encoders (20) | Training | 0.798 | 0.151 | 0.760 | 0.139 |
| | Linear PCA encoders (20) | Validation | 0.787 | 0.017 | 0.737 | 0.165 |
| | Linear PCA encoders (20) | Over-fitting rate | 8.553 | 1.009 | 1.398 | 1.641 |
| | Linear PCA encoders (20) (+FFT) | Training | 0.808 | 0.151 | 0.770 | 0.142 |
| | Linear PCA encoders (20) (+FFT) | Validation | 0.802 | 0.017 | 0.755 | 0.169 |
| | Linear PCA encoders (20) (+FFT) | Over-fitting rate | 8.507 | 0.999 | 1.385 | 1.633 |

Table S8: Summary of statistical values for exploration properties with different strategies on enzyme family classification

| Task | Strategy | Performance | Max value | Min value | Average | Standard deviation |
|---|---|---|---|---|---|---|
| Protein folding classification | Only properties | Training | 0.824 | 0.757 | 0.795 | 0.009 |
| | Only properties | Validation | 0.818 | 0.743 | 0.774 | 0.014 |
| | Only properties | Over-fitting rate | 1.089 | 0.951 | 1.026 | 0.022 |
| | Only properties (+FFT) | Training | 0.832 | 0.757 | 0.811 | 0.009 |
| | Only properties (+FFT) | Validation | 0.840 | 0.753 | 0.800 | 0.015 |
| | Only properties (+FFT) | Over-fitting rate | 1.079 | 0.960 | 1.014 | 0.021 |
| | Linear PCA encoders (20) | Training | 0.802 | 0.119 | 0.753 | 0.145 |
| | Linear PCA encoders (20) | Validation | 0.800 | 0.006 | 0.732 | 0.167 |
| | Linear PCA encoders (20) | Over-fitting rate | 19.720 | 0.990 | 1.956 | 4.075 |
| | Linear PCA encoders (20) (+FFT) | Training | 0.828 | 0.119 | 0.778 | 0.151 |
| | Linear PCA encoders (20) (+FFT) | Validation | 0.828 | 0.006 | 0.764 | 0.174 |
| | Linear PCA encoders (20) (+FFT) | Over-fitting rate | 19.720 | 0.971 | 1.946 | 4.077 |

Table S9: Summary of statistical values for exploration properties with different strategies on protein folding classification

Finally, as shown in the previous tables, we compare the performances obtained using the encoders proposed in this work with the different physicochemical properties reported in the AAIndex database with various usage strategies. Table S11 shows the results of the comparison, evaluating the maximum values in each of the proposed strategies of use of the physicochemical properties and the average of the executions generated in the process of training the coders proposed in this work. As can be seen in Table S11, our

| Task | Strategy | Performance | Max value | Min value | Average | Standard deviation |
|---|---|---|---|---|---|---|
| Function classification | Only properties | Training | 0.878 | 0.864 | 0.871 | 0.002 |
| | Only properties | Validation | 0.870 | 0.851 | 0.862 | 0.002 |
| | Only properties | Over-fitting rate | 1.025 | 0.997 | 1.010 | 0.004 |
| | Only properties (+FFT) | Training | 0.887 | 0.859 | 0.877 | 0.00 |
| | Only properties (+FFT) | Validation | 0.886 | 0.853 | 0.875 | 0.003 |
| | Only properties (+FFT) | Over-fitting rate | 1.017 | 0.991 | 1.002 | 0.003 |
| | Linear PCA encoders (20) | Training | 0.876 | 0.336 | 0.844 | 0.116 |
| | Linear PCA encoders (20) | Validation | 0.865 | 0.108 | 0.823 | 0.164 |
| | Linear PCA encoders (20) | Over-fitting rate | 3.106 | 1.004 | 1.116 | 0.456 |
| | Linear PCA encoders (20) (+FFT) | Training | 0.878 | 0.336 | 0.848 | 0.117 |
| | Linear PCA encoders (20) (+FFT) | Validation | 0.880 | 0.108 | 0.838 | 0.167 |
| | Linear PCA encoders (20) (+FFT) | Over-fitting rate | 3.106 | 0.994 | 1.103 | 0.459 |

Table S10: Summary of statistical values for exploration properties with different strategies on protein function classification

encoders reach higher performance in the tasks of classifying proteins of interaction with DNA and the prediction of enzymatic families. However, the average performance in folding and function classification cases is slightly lower. Nonetheless, this may be mainly due to a favourable split in the dataset preparation stage; the maximum performance achieved by our encoders in the training stage was much higher (0.92 and 0.99, respectively).

| Task | Performance | Our Encoders | Comparison with physicochemical properties | | | |
|---|---|---|---|---|---|---|
| | | Average process | Max Property | Max FFT(Property) | Max PCA(Property) | Max FFT(PCA) |
| DNA-Binding protein classification | Training | 0.824 ± 0.014 | 0.671 | 0.675 | 0.664 | 0.654 |
| | Validation | 0.842 ± 0.082 | 0.711 | 0.713 | 0.709 | 0.688 |
| Enzyme family classification | Training | 0.863 ± 0.037 | 0.801 | 0.816 | 0.798 | 0.808 |
| | Validation | 0.862 ± 0.059 | 0.797 | 0.809 | 0.787 | 0.802 |
| Folding classification | Training | 0.809 ± 0.011 | 0.824 | 0.832 | 0.802 | 0.828 |
| | Validation | 0.809 ± 0.048 | 0.818 | 0.840 | 0.800 | 0.828 |
| Function classification | Training | 0.820 ± 0.042 | 0.878 | 0.887 | 0.876 | 0.878 |
| | Validation | 0.850 ± 0.072 | 0.870 | 0.886 | 0.865 | 0.880 |

Table S11: Comparison performance between our encoders and different strategies to use physicochemical properties to encode protein sequences

# S5 Exploring biological activities for peptide sequences

To demonstrate the possible uses of the encoders proposed in this work to design and explore new sequences, predictive models for the classification of biological activity of peptide sequences were developed using the Random Forest algorithm as a supervised learning method. In addition, new sequences were evaluated to determine the probability that they will be classified correctly within the current knowledge of the model. Figure S1 illustrates the proposed methodology for the entire sequence exploration analysis, which includes four stages that will be explained below.

## S5.1 Processing peptide sequences

The collection of sequences to assemble the data sets was developed from the Peptipedia database, obtaining all the sequences classified as antimicrobial and all the sequences that are not antimicrobial— approximately 30,000 for each category. Then, sequences with antifungal, antibacterial, anti-cancer, anti-viral, and anti-HIV activities were filtered from the antimicrobial sequences, which were found in similar proportions. It is essential to mention that approximately 30% of the filtered sequences had a moonlight character. That is, they have been reported with more than one activity simultaneously. Finally, from the separations generated, the data sets for training, validation, and use for exploration are generated, with a total of 40,000 to train, 10,000 to validate, and 10,000 to explore, generating binary data sets for the case of classification of antimicrobial peptides and multilabel in the case of subcategories of antimicrobial peptides.

## S5.2 Training classification models

The training of predictive models was based on the use of the training and validation data sets:

1. All the sequences were encoded following the protocols developed in this work, using the eight multi-property encoders.
2. The models are trained by applying Random Forest algorithms and following a cross-validation strategy to prevent overfitting. The algorithm configuration hyperparameters are left by default as established in the *DMAKit tool*[Medina-Ortiz et al.(2020)Medina-Ortiz, Contreras, Quiroz, Asenjo, and Olivera-Nappa].
3. Model performance is measured based on the classification accuracy metrics.
4. The models are exported in *joblib* format to be used

## S5.3 Developing latent statistic space

Once the models were generated, the process of developing latent statistical spaces began. The basic concept of this methodology is to focus on the current knowledge of the model to evaluate new sequences of interest. To do this, the numerical representations generated using each of our encoders are used, and the following steps are developed:

Let $M\{R_{P_i}\}$ be the matrix of representations of peptide sequences generated from the encoder based on the physicochemical property $P_i$ with dimension $n \times m$ where $n$ represents the number of sequences and $m$ the number of points of the coding vector. In this case, we set $m = 64$ due to the applicable rules of FFT, where the input must be approximated to the nearest power of two to increase performance. The result corresponds to a vector of size $\frac{l}{2}$ with $l = 128$ due to the size of the sequences.

For each column $V$ of the matrix $M\{R_{P_i}\}$, we estimate a confidence interval at 95% ($CI_95$), which is used as input to generate the latent space of property $P_i$, $S_{P_i}$.

Then, we evaluate whether a new sequence $N$, encoded under the property $P_i$, belongs to the latent space $S_{P_i}$ by checking that each point $j$ in the sequence $N$ falls within the confidence interval $CI_95$ corresponding to that column index. For this, we use a binary classification; 0 if it belongs and 1 if it does not. In this way, once the analysis is finished, a distribution $D(N)$ is generated for the evaluated sequences, which is assumed $\sim binomial_{(n,p)}$ where $n =$ $number\ of\ examples$ and $p = success\ rate$, from which the probability that the sequence $N$ $(pbb(N)_i)$ belongs to the space $S_{P_i}$ of property $P_i$ is estimated.

The previous point is applied to each of the eight encoders. In this way, eight independent probabilities are combined in a single indicator, which is obtained from the multiplication of the $pbb(N)_i$ for the sequence $N$.

### S5.4 Exploring new peptide sequences

New sequences are explored using the latent statistical space described in the previous section. If the sequence exceeds a threshold $t$, the predictive models for classifying microbial activity are applied; if they are classified as AMP, the multilabel model is applied, and one of the AMP subcategories is obtained. In this case, we used a $t = 0.9$, and a total of 10,000 sequences were analyzed.

| **Biological activity** | **Number of sequences** | **Accuracy (%)** |
|---|---|---|
| Antimicrobial | 3513 | 98.1% |
| Anti-viral | 1210 | 87.4% |
| Anti-bacterial | 1350 | 86.1% |
| Anti-cancer | 1980 | 79.5% |
| Anti-fungal | 832 | 74.8% |
| Anti-HIV | 1127 | 79.8% |

Table S12: Performance (accuracy) obtained for the explored biological activity strategies developed on this work

# References

[Cadet et al.(2018a)Cadet, Fontaine, Li, Sanchis, Chong, Pandjaitan et al.] Cadet, F., Fontaine, N., Li, G., Sanchis, J., Chong, M. N. F., Pandjaitan, R., et al. (2018a). A machine learning approach for reliable prediction of amino acid interactions and its application in the directed evolution of enantioselective enzymes. *Scientific reports* 8, 1–15

[Cadet et al.(2018b)Cadet, Fontaine, Vetrivel, Chong, Savriama, Cadet et al.] Cadet, F., Fontaine, N., Vetrivel, I., Chong, M. N. F., Savriama, O., Cadet, X., et al. (2018b). Application of fourier transform and proteochemometrics principles to protein engineering. *BMC bioinformatics* 19, 382

[Kieslich et al.(2021)Kieslich, Alimirzaei, Song, Do, and Hall] Kieslich, C. A., Alimirzaei, F., Song, H., Do, M., and Hall, P. (2021). Data-driven prediction of antiviral peptides based on periodicities of amino acid properties. In *Computer Aided Chemical Engineering* (Elsevier), vol. 50. 2019–2024

[Medina-Ortiz et al.(2020)Medina-Ortiz, Contreras, Quiroz, Asenjo, and Olivera-Nappa] Medina-Ortiz, D., Contreras, S., Quiroz, C., Asenjo, J. A., and Olivera-Nappa, Á. (2020). Dmakit: A user-friendly web platform for bringing state-of-the-art data analysis techniques to non-specific users. *Information systems* 93, 101557

[Siedhoff et al.(2021)Siedhoff, Illig, Schwaneberg, and Davari] Siedhoff, N. E., Illig, A.-M., Schwaneberg, U., and Davari, M. D. (2021). Pypef—an integrated framework for data-driven protein engineering. *Journal of Chemical Information and Modeling* 61, 3463–3476

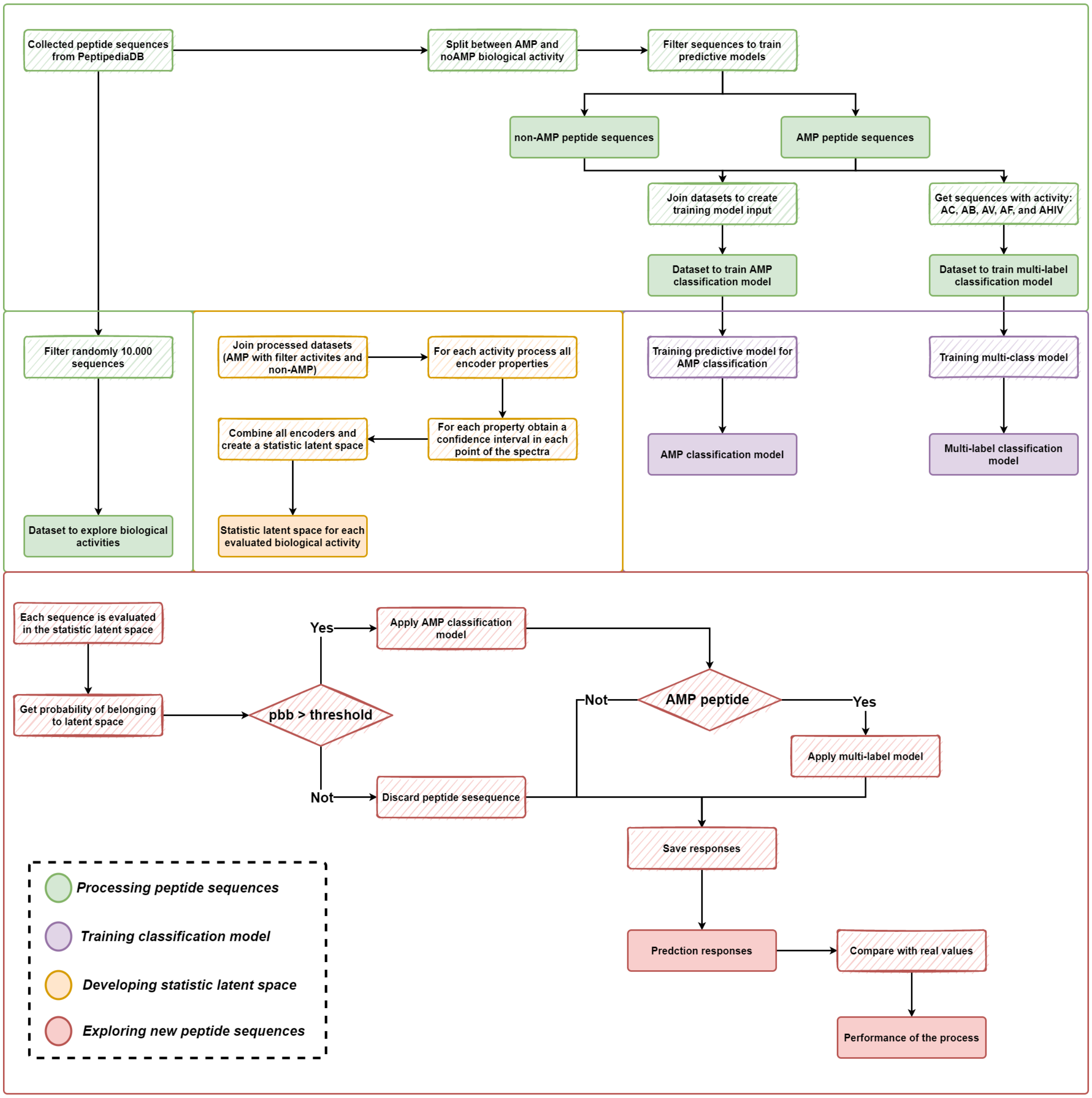


Supplementary Figure S1: Representative schematic of the sequence exploration methodology evaluated by statistical latent space analysis. The four main points are considered, associated with data processing, the training of predictive models, the development of latent statistical spaces, and finally, the application of the components to explore new sequences.
